# Site-Specific Nanoindentation Hardness of Human Root Cementum and Corresponding Alveolar Cortical Bone: Associations with Uncalibrated CBCT and Micro-CT Gray-Value Indices

**DOI:** 10.3390/dj14100670

**Published:** 2026-10-14

**Authors:** Ayano Egashira, Kaori Amano, Tatsuo Yamamoto, Iwao Hasegawa, Kimiko Nakagawa, Yusuke Kozai, Masahiro Izumi, Hanae Shiga, Masahiro Takahashi, Heetae Park, Reina Hatanaka, Tetsutaro Yamaguchi

**Affiliations:** 1 Department of Orthodontics, School of Dentistry, Kanagawa Dental University, 82 Inaoka-cho, Yokosuka 238-8580, Japan; takahashi.masahiro@kdu.ac.jp (M.T.); park@kdu.ac.jp (H.P.); hatanaka@kdu.ac.jp (R.H.); t.yamaguchi@kdu.ac.jp (T.Y.); 2 Department of Anatomy, Graduate School of Dentistry, Kanagawa Dental University, 82 Inaoka-cho, Yokosuka 238-8580, Japan; amano@kdu.ac.jp; 3 Department of Preventive Dentistry and Dental Public Health, Kanagawa Dental University, 82 Inaoka-cho, Yokosuka 238-8580, Japan; yamamoto.tatsuo@kdu.ac.jp; 4 University Center of Legal Medicine, Kanagawa Dental University, 82 Inaoka-cho, Yokosuka 238-8580, Japan; i.hasegawa@kdu.ac.jp (I.H.); nakagawa@kdu.ac.jp (K.N.); 5 Department of Education Planning, Kanagawa Dental University, 82 Inaoka-cho, Yokosuka 238-8580, Japan; kozai@kdu.ac.jp (Y.K.); h.shiga@kdu.ac.jp (H.S.); 6 Department of Diagnostic Imaging, Graduate School of Dentistry, Kanagawa Dental University, 82 Inaoka-cho, Yokosuka 238-8580, Japan; m.izumi@kdu.ac.jp

**Keywords:** root cementum, alveolar cortical bone, nanoindentation, cone-beam computed tomography, micro-computed tomography, periodontal supporting tissues, mechanical properties

## Abstract

**Background/Objectives**: The local mechanical properties of periodontal supporting tissues influence their responses to orthodontic forces. This exploratory study sought to evaluate site-specific nanoindentation hardness in human root cementum and adjacent alveolar cortical bone and examine its association with cone-beam computed tomography (CBCT)- and micro-computed tomography (micro-CT)-derived relative imaging indices. **Methods**: Specimens from seven human cadavers were analyzed. Indentation hardness (HIT) was measured in the central and apical regions of root cementum and adjacent alveolar cortical bone. CBCT gray values and micro-CT-derived relative imaging indices were obtained from anatomically matched regions without phantom-based mineral-density calibration. Nonparametric tests, multiple linear regression analyses, and Spearman’s rank correlation analyses were performed. **Results**: Root cementum exhibited significantly higher HIT in the central region than in the apical region (*p* < 0.001). The central region and mandibular location were independently associated with higher cementum HIT. No significant regional variation or associated factors were identified for alveolar cortical bone. Neither CBCT nor micro-CT-derived indices were significantly associated with local tissue hardness. **Conclusions**: Human root cementum exhibits site-specific variation in hardness, unlike the adjacent alveolar cortical bone. Uncalibrated CT-derived relative imaging indices may not be significantly associated with local tissue hardness.

## 1. Introduction

Craniofacial bones, essential in mastication, swallowing, and speech, are continuously subjected to functional loading [1]. Depending on the anatomical location, loading environment, and remodeling history, the material properties of bone are not uniform and vary even within cortical bone [2]. The mechanical properties of bone, such as stiffness and hardness, are also influenced by its structural organization and microdamage repair processes, as it is a hierarchical composite material composed of minerals, collagen, and water [3,4,5]. In addition, age-related changes in cellular activity and mineralization patterns affect the mechanical properties of bone [6,7]. These findings highlight the importance of evaluating bone tissue at the local level rather than treating it as a homogeneous material [2].

Traditionally, the mechanical properties of bone have been assessed using macroscopic testing; however, such approaches have limitations because of the inherent heterogeneity of bone at the microstructural level [8]. Nanoindentation has been widely used to evaluate this heterogeneity, enabling the measurement of hardness and the elastic modulus at the lamellar level and revealing substantial site-specific and interindividual variations [9,10]. Hardness is an indicator of resistance to microdeformation and may reflect the local mechanical properties [11]. Furthermore, hardness is closely related to mineralization, and differences in mineral content have been reported to contribute to variations in the elastic modulus and strength [12,13,14,15]. These observations support the need to evaluate bone material properties at anatomically localized levels rather than relying on average values [16,17,18].

In alveolar cortical bone-supporting dentition, the cortical bone adjacent to the tooth root is considered a mechanically important region responsible for transmitting masticatory and orthodontic forces. The tooth root surface (cementum and dentin) and the adjacent cortical bone are anatomically contiguous but differ in composition and remodeling patterns, suggesting differences in local mechanical properties. These differences may influence stress distribution, microdamage formation, and tissue responses [19]. Furthermore, mineralized tissues, such as bone and dentin, possess spatially varying material properties to optimize mechanical function [9,10,20].

Direct site-matched comparisons of root cementum and anatomically adjacent alveolar cortical bone within the same human specimen are scarce [19,21]. Previous nanoindentation studies have primarily evaluated individual mineralized tissues or long bones, and relatively few studies have simultaneously examined tissue-specific and regional hardness within the human dentoalveolar complex [5,9,10,18,22,23]. Furthermore, whether imaging-derived indices obtained from anatomically matched regions correspond to directly measured tissue-level hardness remains to be determined. Therefore, the combined assessment of root cementum, adjacent alveolar cortical bone, and site-matched CT-derived indices may provide information that cannot be obtained by evaluating each tissue or imaging modality separately.

CT-derived density indices have attracted attention as potential indicators of bone density and mechanical properties [24]. In conventional medical CT, HU values correlate with bone density and compressive strength [25]. However, in cone-beam computed tomography (CBCT), which is widely used in dentistry, quantitative accuracy remains limited owing to variability in imaging conditions and device-specific characteristics [26,27]. Although CBCT-derived gray values can potentially facilitate bone quality assessment, the extent to which these imaging-derived indices correspond to actual local material properties, such as hardness measured by nanoindentation, remains unclear [28]. CBCT gray values and micro-CT gray-scale indices cannot necessarily be interpreted as absolute mineral-density values unless acquisition-specific calibration is performed. Therefore, when phantom-based calibration is unavailable, their relationship with local mechanical properties should be evaluated as the correspondence between relative imaging indices and directly measured hardness.

This exploratory study was conducted to compare the nanoindentation hardness of human root cementum and anatomically adjacent alveolar cortical bone by tissue type and root region, and determine whether CBCT- and micro-CT-derived relative imaging indices obtained from matched regions were associated with local hardness. We hypothesized that hardness would differ according to tissue type and anatomical region and that uncalibrated relative imaging indices would show limited correspondence with tissue-level hardness under the present acquisition conditions.

## 2. Materials and Methods

### 2.1. Cadaveric Specimens and Ethical Approval

Human skeletal specimens donated to our institution were used for dental education and research purposes in accordance with relevant domestic regulations. Cadavers were fixed in 10% formalin and stored in 50% ethanol. Written informed consent was obtained from the donors’ families. The study protocol was originally approved by the Research Ethics Committee of Kanagawa Dental University on 9 January 2024 (Approval No. 975). A subsequent amendment concerning the addition of research collaborators and the updating of education and training completion dates was approved on 26 August 2026 (Approval No. 1226). The study was performed in accordance with the approved research protocol. Jawbones and extracted teeth were obtained from seven cadavers used for anatomical training. Due to the limited availability of human cadaveric jaw specimens suitable for anatomically matched nanoindentation and CT analyses, the study was designed as an exploratory investigation. Maxillary and mandibular specimens from each cadaver were prepared and analyzed. Details of the cadavers are presented in Table 1. After separation of the maxilla and mandible, the soft tissues were carefully removed. Tooth–alveolar bone blocks, preserving the anatomical relationship between the teeth and the surrounding alveolar cortical bone, were harvested from the cadaveric jaws using a diamond disk (Diamond Disk, Shofu, Kyoto, Japan). Specimens with missing teeth were excluded. Cross-sectional maxillary and mandibular specimens were subsequently prepared from each cadaver.

### 2.2. Specimen Preparation

**Figure 1:**
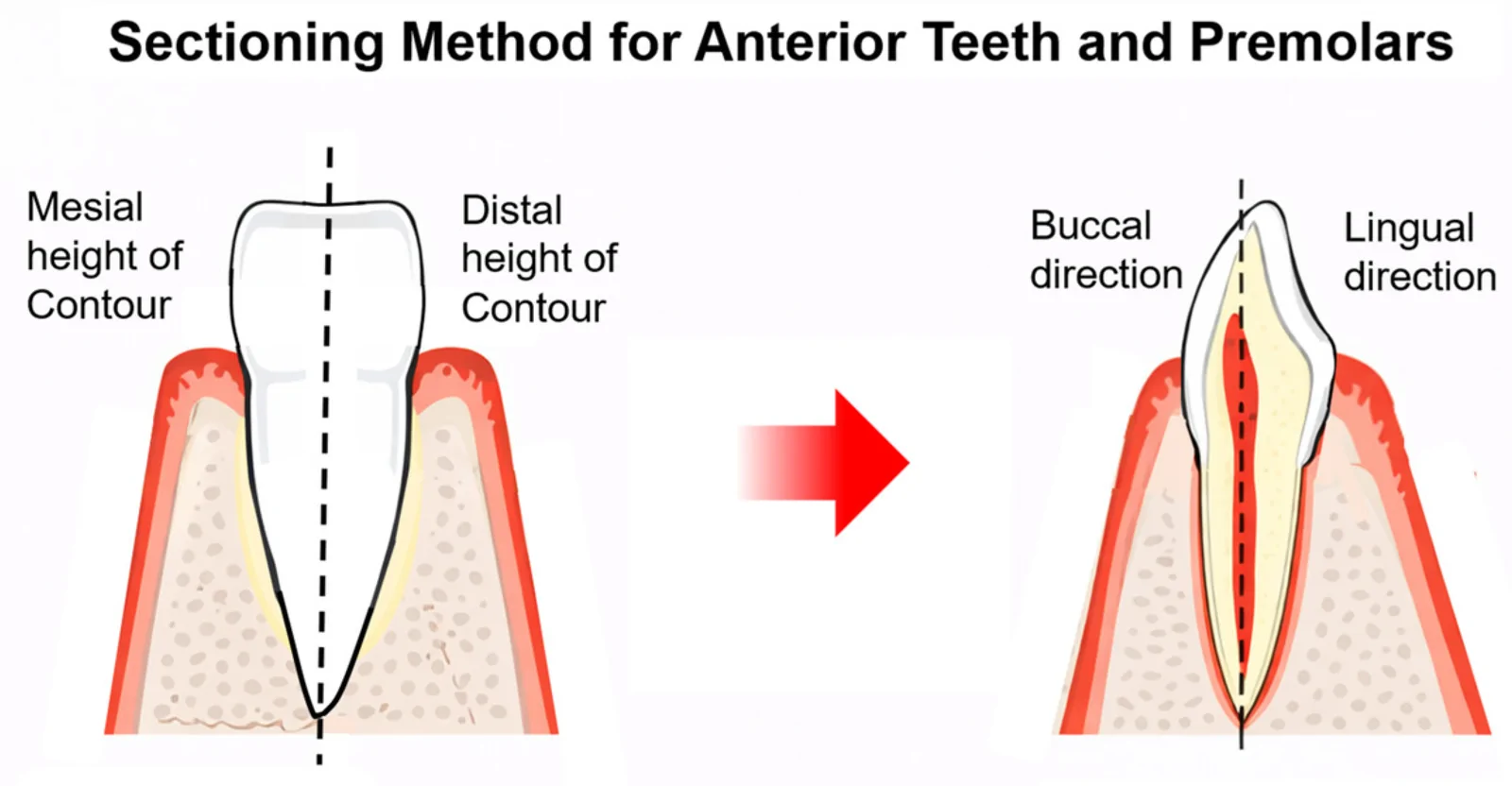
Schematic illustration of the sectioning procedure used for anterior teeth and premolars. Specimens were sectioned along the buccolingual direction through the center of the tooth crown and root to obtain slices that included the cementum and adjacent alveolar cortical bone. Mesial and distal heights of contour are indicated. The dashed lines indicate the sectioning planes.

**Figure 2:**
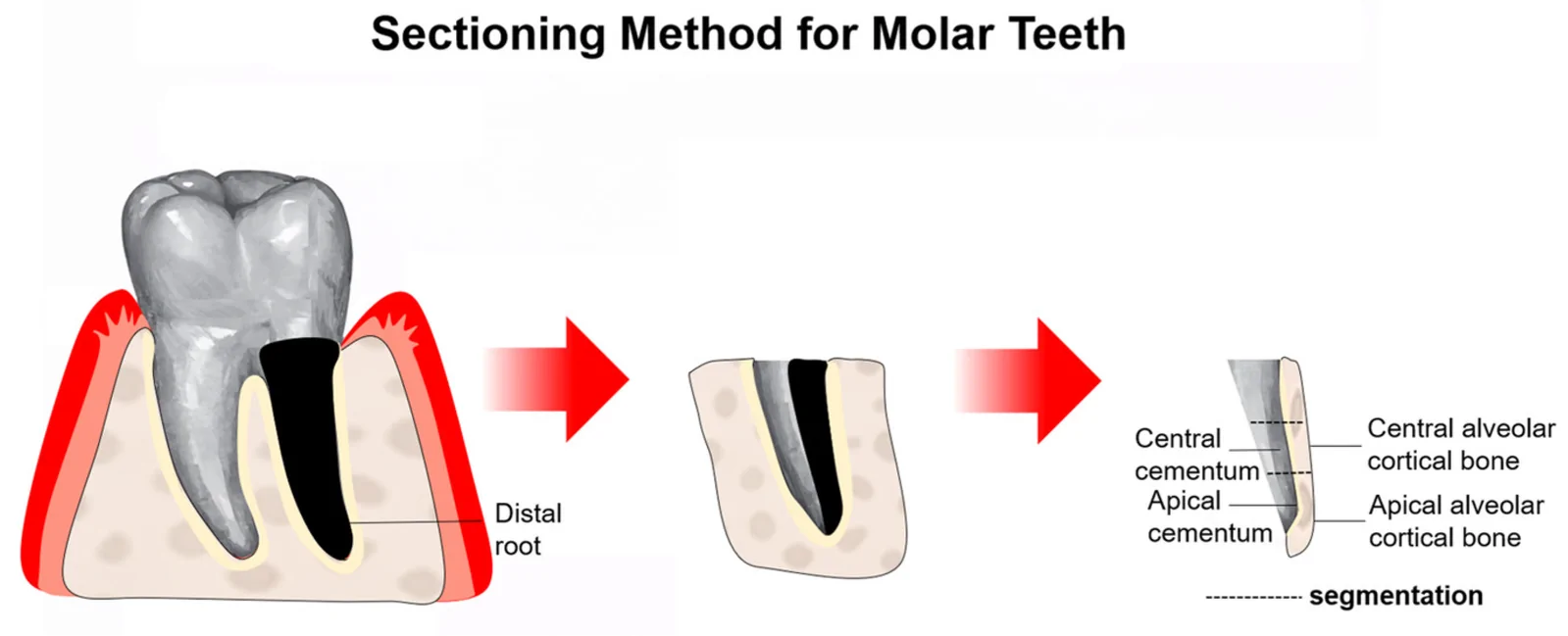
Schematic illustration of the sectioning procedure used for molar teeth. The distal root was selected and sectioned to obtain specimens including the middle and apical cementum as well as the corresponding alveolar cortical bone regions for analysis.

The tooth sectioning method adopted in this study is illustrated in Figure 1 and Figure 2. The cutting direction and site selection were determined based on previously reported methods for evaluating the root cementum and region-specific mechanical properties of dental hard tissues [29,30], with modifications to enable anatomical comparison in the present study. After separating the crowns and roots, specimens were sectioned longitudinally along the tooth axis. For incisors, canines, and single-rooted premolars, sections were made along the planes connecting the midpoint of the maximum crown contour to the root apex on both the buccal and lingual sides. For multi-rooted premolars, sections were made along planes connecting the midpoint of the maximum crown contour to each root apex. For molars, the distal root was first separated, and each root was subsequently sectioned longitudinally along the tooth axis. This protocol was designed to standardize anatomical positioning and ensure reproducibility of measurement sites.

Each segment was embedded in epoxy resin. The embedded blocks were then ground using a motorized polishing wheel (APU-138P, Refine Tec, Yokohama, Kanagawa, Japan) with interchangeable silicon carbide grinding discs of diminishing grit size (400, 1000, and 2000 grit). Between each grinding step and after polishing, sample surfaces were rinsed with running water to remove debris and then air-dried. To visualize tooth morphology, images of each sectioned surface were captured using a digital microscope (VHX-6000, KEYENCE, Osaka, Japan) at 20× magnification.

### 2.3. Nanoindentation Testing

To evaluate the mechanical properties of teeth and alveolar cortical bone, nanoindentation testing was performed using a nanoindenter (ENT-1100a, Elionix, Tokyo, Japan) equipped with a Berkovich indenter tip angle (α = 65.03°). The testing conditions were set at a maximum load of 100 mN, a holding time of 30 s, and a minimum spacing of approximately 0.1 mm between indentations. All specimens were polished to a thickness of approximately 10 mm using 400-grit sandpaper prior to testing.

### 2.4. CBCT and Micro-CT Image Acquisition

CT imaging was performed to obtain relative imaging indices from root cementum and alveolar cortical bone. Micro-CT images were obtained using a ScanXmate-L080 system (Comscan Tecno Co., Ltd., Yokohama, Kanagawa, Japan) operated at 65 kV and 63 μA. Images were reconstructed using coneCTexpress (ver. 1.82, Comscan Tecno Co., Ltd., Yokohama, Kanagawa, Japan). The detector pixel size was 100 μm, the geometric magnification was 2.00225, and the resulting reconstructed spatial sampling interval was approximately 49.944 μm. The reconstructed images were exported as 16-bit TIFF files and analyzed using relative gray-scale indices. No hydroxyapatite phantom or other reference standard was used to convert the gray-scale values into absolute mineral-density values. No normalization across specimens was performed; therefore, the micro-CT values were interpreted as relative imaging indices rather than absolute mineral-density measurements.

CBCT imaging was performed using 3D Accuitomo (J. Morita, Kyoto, Japan) with the following parameters: tube voltage 90.0 kV, tube current 5.0 mA, slice thickness 0.48 mm, field of view 60 × 60 mm, and exposure time 17.5 s; native in-plane pixel spacing, 0.160 × 0.160 mm; and slice interval, 0.480 mm, as confirmed from the original DICOM metadata. CBCT gray values were treated as device- and acquisition-dependent relative values and were not converted to Hounsfield units or absolute mineral-density values. No phantom-based density calibration was performed. The micro-CT TIFF images and CBCT DICOM datasets were imported into OsiriX MD (Pixmeo, Geneva, Switzerland) for image display, anatomical matching, and region-of-interest analysis.

### 2.5. Definition of Measurement Regions and ROI Placement

**Figure 3:**
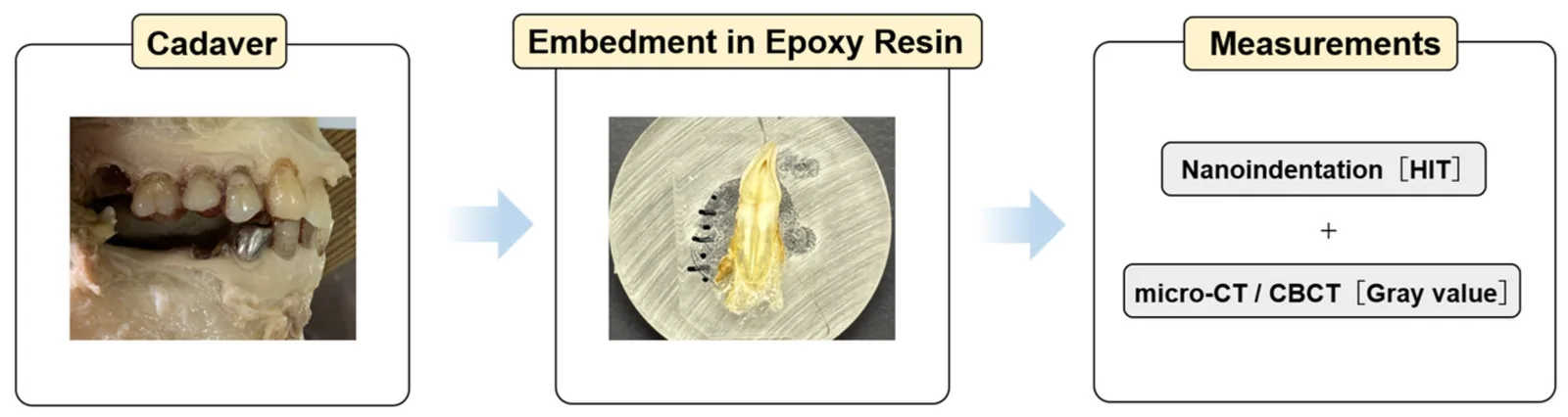
Schematic overview of the experimental workflow. Teeth and surrounding alveolar bone specimens obtained from cadavers were embedded in epoxy resin. Mechanical properties (indentation hardness [HIT]) were evaluated via nanoindentation testing, and imaging-derived indices were obtained from micro-computed tomography (micro-CT) and cone-beam computed tomography (CBCT) for subsequent analysis.

**Figure 4:**
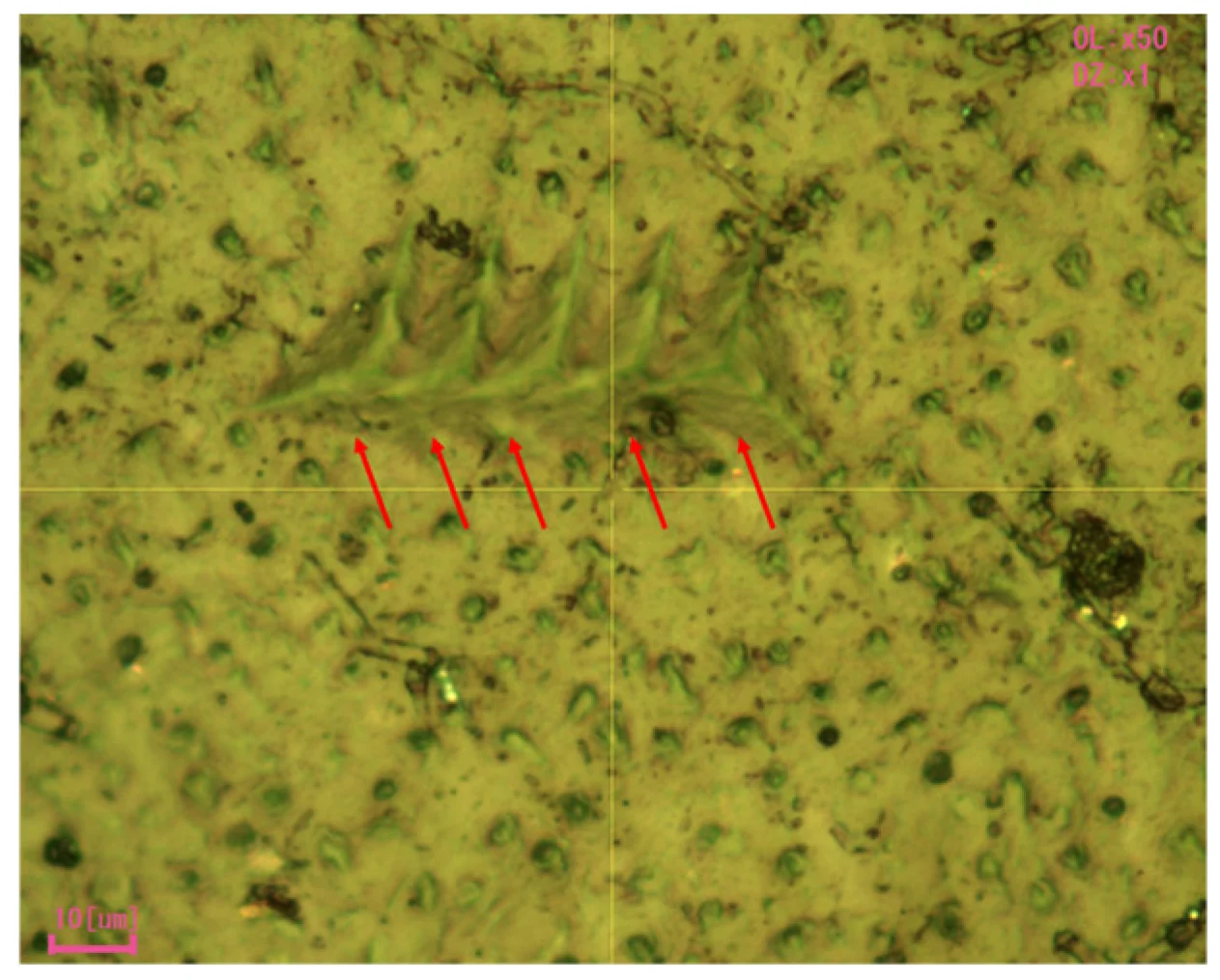
Representative nanoindentation image of the root cementum. Five indentation sites were created within the measurement region, and the red arrows indicate the individual indentation marks used for hardness measurements.

The overall workflow of specimen preparation, nanoindentation testing, and imaging analysis is illustrated in Figure 3. Each specimen was divided into three regions along the tooth axis: cervical, central, and apical regions. The distance from the cementoenamel junction (CEJ) to the root apex was divided into three equal parts; the middle third was defined as the central region, and the apical third as the apical region. The central and apical regions were used for the nanoindentation and CT analyses. Indentations were performed within the cementum layer while avoiding the cementum–dentin junction whenever possible. Measurement points were defined along a line perpendicular to the longitudinal axis within each region. For root cementum, measurements were performed on the cementum surface facing the alveolar cortical bone. For alveolar cortical bone, measurements were performed on the cortical bone surface facing the root cementum. In the nanoindentation test, five measurements were obtained for each of the following regions: the middle and apical regions of the root cementum, and the middle and apical regions of the alveolar cortical bone; the average hardness was calculated using the analysis software supplied with the instrument [31]. Representative indentation sites used for the five measurements are shown in Figure 4. Five indentation attempts were performed at each measurement site. When valid hardness values were obtained, the mean of the valid measurements was used for analysis. Measurement sites were excluded only when no valid hardness value could be obtained from any of the five indentation attempts. The nanoindenter was calibrated according to the manufacturer’s recommendations before testing. All nanoindentation tests were performed under a peak load of 100 mN using a Berkovich indenter [32]. The corresponding maximum indentation depth was recorded automatically by the instrument.

### 2.6. ROI Placement and Image Analysis

**Figure 5:**
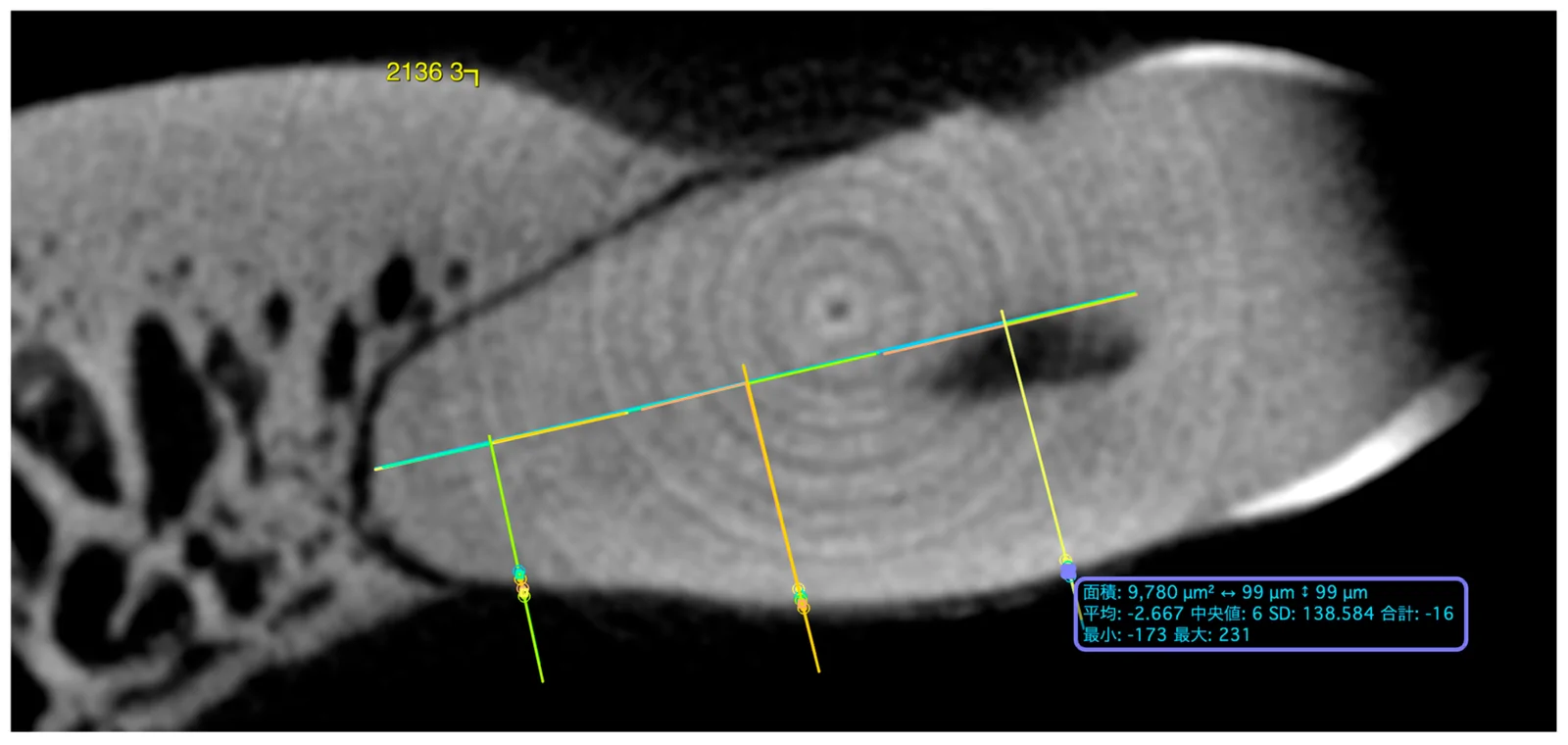
Representative CBCT image showing the placement of regions of interest (ROIs) for imaging analysis. ROIs were positioned at the anatomically matched central and apical regions of the root cementum and adjacent alveolar cortical bone. The yellow lines indicate the boundaries dividing the tooth root into three equal regions: cervical, central, and apical. For the representative ROI shown, the measured area was 9780 µm^2^, and the CBCT gray-value statistics were as follows: mean, −2.667; median, 6; standard deviation (SD), 138.584; sum, −16; minimum, −173; and maximum, 231.

For imaging analysis, two-dimensional rectangular ROIs, approximately 90–100 × 90–100 μm in physical dimensions, were manually placed at anatomically matched central and apical measurement sites on reconstructed cross-sectional images displayed in OsiriX MD. The ROIs were defined on the basis of physical dimensions rather than a fixed number of pixels or voxels. ROI placement was guided by anatomical landmarks, the corresponding sectioned-surface images, and gray-value line profiling. Gray-value line profiles were used to identify local changes in image intensity corresponding to the target tissue region. For cementum measurements, each ROI was positioned within the target layer while minimizing, as far as possible, the inclusion of adjacent dentin and surrounding tissues. The locations of the ROIs used for CBCT and micro-CT measurements are illustrated in Figure 5. Because the ROIs were defined according to their physical dimensions rather than a predetermined number of pixels or voxels, the number of image samples contributing to individual ROIs and their positions relative to the reconstructed image grid were not explicitly standardized. The nominal ROI dimension of approximately 90–100 μm was smaller than the native in-plane pixel spacing of CBCT (160 μm), whereas it corresponded to approximately two reconstructed sampling intervals for micro-CT (approximately 49.944 μm). Therefore, ROI-derived statistics, particularly for CBCT, should not be interpreted as measurements obtained from a corresponding number of independent native image samples. The same nominal physical ROI dimensions were used for both imaging modalities to target anatomically corresponding regions; however, the number of image samples contributing to each ROI differed according to the spatial sampling characteristics of each modality. Mean and maximum gray-value indices were calculated from the image samples included within each ROI. Nanoindentation measurements were excluded when valid hardness values could not be obtained. CT measurements were excluded when CBCT gray values or micro-CT relative imaging indices could not be determined within the predefined ROI. The number of observations available for each analysis is reported in the corresponding tables. Because individual technical failures and exclusions were not systematically recorded in sufficient detail during data acquisition, the exact number of excluded measurements and the specific reason for each exclusion could not be reliably reconstructed retrospectively. Therefore, no estimated exclusion counts were reported. Since no phantom-based mineral-density calibration was performed, all CBCT and micro-CT measurements were interpreted as relative imaging indices rather than quantitatively calibrated mineral-density values.

### 2.7. Statistical Analysis

To evaluate local hardness characteristics (indentation hardness [HIT], MPa) in root cementum and alveolar cortical bone, regional differences, inter-tissue differences, associated factors, and relationships with imaging-derived indices were analyzed. Regional differences in HIT were assessed using the Wilcoxon signed-rank test for paired comparisons between central and apical regions within each tissue. Inter-tissue comparisons between cementum and alveolar cortical bone were performed separately for each region using the Wilcoxon signed-rank test.

To identify factors associated with HIT, univariate analyses were conducted using jaw (maxilla/mandible), tooth type, presence of metal prostheses, presence of opposing tooth, root canal treatment, and individual variation as explanatory variables. The Mann–Whitney *U* test was used for comparisons between two groups, whereas the Kruskal–Wallis test was used for comparisons among three or more groups. All analyses were stratified by tissue type and region.

Variables with *p* < 0.20 in univariate analyses were selected as candidate predictors and included in multiple linear regression models with HIT as the dependent variable. Separate models were constructed for cementum and alveolar cortical bone, with all variables entered simultaneously. Unstandardized regression coefficients (B), 95% confidence intervals, and *p* values were calculated. Model fit was evaluated using the adjusted *R*^2^ and overall model significance. Multiple teeth were obtained from the same cadaver; therefore, specimen ID was included as a categorical covariate in the regression models to partially account for inter-individual variation. However, the models were not multilevel or mixed-effects models and therefore may not have fully accounted for within-cadaver clustering. Because Specimen 2 had a history of multiple myeloma and contributed a relatively large number of teeth to the analyses, sensitivity analyses were additionally performed after excluding all measurements derived from this donor. The primary analyses were repeated using the remaining six cadavers to assess the robustness of the findings to the inclusion of Specimen 2. To ensure consistency throughout the study, all nanoindentation measurements and image analyses were conducted by a single examiner.

To assess the relationship between imaging-derived indices and material properties, correlations between CBCT-derived gray values, micro-CT-derived relative imaging indices, and HIT were evaluated using Spearman’s rank correlation coefficient. Since 16 correlation tests were performed, Bonferroni correction was applied, and the corrected significance threshold was set at α = 0.003125. Statistical analyses were performed using IBM SPSS Statistics version 28.0.1 (IBM Corp., Armonk, NY, USA), with a significance level set at 5%. Schematic illustrations and final figure assembly and layout were prepared using Microsoft PowerPoint (Microsoft Corporation, Redmond, WA, USA).

## 3. Results

### 3.1. Characteristics of the Study Sample

The characteristics of the study sample are summarized in Table 1. The study included specimens from seven cadavers (one male and six females), with ages ranging from 81 to 94 years. The number of teeth analyzed per cadaver for nanoindentation, CBCT, and micro-CT ranged from 13 to 27.

### 3.2. Regional Differences in Indentation Hardness and CT-Derived Imaging Indices

For regional comparisons, the median HIT in root cementum was significantly higher in the central region (205.8 MPa) than in the apical region (157.7 MPa) (*p* < 0.001; Table 2). In contrast, no significant difference was observed in alveolar cortical bone, with median HIT values of 107.8 MPa in the central region and 113.0 MPa in the apical region, respectively (*p* = 0.283; Table 2). Regarding imaging-derived indices in root cementum, all CBCT and micro-CT parameters were significantly higher in the central region than in the apical region (all *p* < 0.001). Similarly, in alveolar cortical bone, all imaging indices were significantly higher in the central region (*p* = 0.003–0.010, Table 2).

For inter-tissue comparisons, in the central region, the HIT of root cementum (median: 206.6 MPa; IQR: 134.3–282.7) was significantly higher than that of alveolar cortical bone (median: 107.8 MPa; IQR: 80.8–156.0) (*p* < 0.001, Table 3). In the apical region, no significant difference was observed between cementum (162.2; IQR: 117.0–226.7) and alveolar cortical bone (113.4 MPa; IQR: 92.2–155.5) (*p* = 0.299). For imaging-derived indices in the central region, all parameters were significantly higher in cementum than in the alveolar cortical bone (all *p* < 0.001). A similar trend was observed in the apical region for all indices, except HIT (all *p* < 0.001, Table 3).

### 3.3. Factors Associated with Indentation Hardness

Univariate analysis revealed a significant difference in HIT between the maxilla and mandible in the central region of the root cementum, with higher values in the mandible (*p* = 0.027, Table S1). The presence of metal prostheses in the apical region of the alveolar cortical bone was significantly associated with higher HIT values (*p* = 0.039). Other variables (specimen ID, tooth type, presence of opposing tooth, and root canal treatment) were not significantly associated with HIT (Table S1). In the multivariate analysis presented in Table 4, which included factors with *p* < 0.200, the central location of the root cementum was significantly associated with an increase in HIT (B = 42.6, 95% CI: 13.3–71.9, *p* = 0.005, Table 4). Furthermore, maxillary location was significantly associated with lower cementum HIT compared with mandibular location in the fitted model (B = −31.6, 95% CI: −61.3 to −1.9, *p* = 0.037). In contrast, specimen ID and root canal treatment were not statistically significant. The cementum model showed limited explanatory power (adjusted *R*^2^ = 0.066), although the overall model reached statistical significance (*p* = 0.003). In contrast, the alveolar cortical bone model showed no significant association with HIT and demonstrated poor model fit (adjusted *R*^2^ = −0.023, *p* = 0.902, Table 4).

### 3.4. Correlations Between Indentation Hardness and CT-Derived Imaging Indices

Spearman’s rank correlation analysis revealed no significant correlations between HIT and CBCT- or micro-CT-derived indices in either the central or apical regions of root cementum (Table S2). In the central region of alveolar cortical bone, weak negative correlations were observed between HIT and the maximum micro-CT index (*r*_s_ = −0.213, *p* = 0.033) and between HIT and the mean micro-CT index (*r*_s_ = −0.235, *p* = 0.018). However, neither association remained statistically significant following Bonferroni correction for multiple comparisons (α = 0.003125). No significant correlations were found with CBCT-derived indices or in the apical region of alveolar cortical bone. Sensitivity analyses excluding Specimen 2 generally yielded results consistent with the primary analyses (Table S3). The higher HIT of central versus apical root cementum, the higher HIT of central root cementum versus adjacent alveolar cortical bone, and the associations of region and jaw with cementum HIT in the multivariable analysis were retained. The absence of significant associations between HIT and CBCT- or micro-CT-derived indices also remained unchanged. However, the comparison between apical root cementum and adjacent alveolar cortical bone changed from non-significant in the primary analysis (*p* = 0.299) to statistically significant after exclusion of Specimen 2 (*p* < 0.001).

## 4. Discussion

Herein, the local material properties of human root cementum and adjacent alveolar cortical bone were directly measured using nanoindentation. Their relationships with imaging-derived indices obtained from CBCT and micro-CT were investigated. The central region of root cementum exhibited significantly higher hardness than the apical region, suggesting clear site-specific characteristics (Table 2). In the central region, cementum hardness was significantly higher than that of alveolar cortical bone (Table 3). Multiple linear regression analysis identified the central region and jaw (maxilla/mandible) as independent factors associated with cementum hardness (Table 4). In contrast, no regional differences or significant associated factors were identified in alveolar cortical bone (Table 2 and Table 4). Furthermore, no significant correlations were observed between HIT and CBCT or micro-CT-derived imaging indices in either tissue (Table S2). These exploratory findings indicate that root cementum and adjacent alveolar cortical bone exhibit different site-specific hardness patterns. Under the present acquisition and analysis conditions, uncalibrated CT-derived relative imaging indices were not significantly associated with local hardness.

Higher hardness levels observed in the central region of root cementum may reflect structural and functional gradients within the periodontal complex. Periodontal tissues consist of cementum, periodontal ligament, and alveolar cortical bone, and continuous transitions have been reported in material properties across their interfaces [33,34,35]. Such gradient structures reduce stress concentration and facilitate efficient load transfer [34,36]. In addition, cementum is a tissue that undergoes continuous apposition throughout life; acellular cementum predominates in the mid-root region, whereas cellular cementum is more abundant in the apical region [37,38]. The differences in hardness between the central and apical regions observed in this study (Table 2) may reflect variations in formation patterns and mineralization. However, as cementum type was not confirmed histologically in the present specimens, this interpretation is speculative. The association between jaw location and cementum hardness in the fitted regression model may reflect differences in local mechanical environments. However, this interpretation remains tentative because of the small number and restricted age range of the cadavers.

In contrast, the absence of significant factors associated with the alveolar cortical bone (Table 4) suggests that its mechanical properties are not governed by a single determinant. Bone mechanical properties rely on multiple factors, including mineral content, organic matrix composition, collagen orientation, microstructure, and water content [39,40,41]. Moreover, three-dimensional microstructural features, such as trabecular architecture and lamellar organization, facilitate load transfer and deformation behavior [42,43,44,45]. Herein, although some differences were observed in univariate analyses (Table S1), no independent factors were identified in multivariate models (Table 4). This finding likely reflects the multifactorial and hierarchical nature of bone material properties. In addition, continuous bone remodeling may result in the coexistence of bone units at different stages of maturation, contributing to spatial and temporal heterogeneity in local material properties [38,46,47].

A key finding of this study was the lack of a significant association between CBCT- and micro-CT-derived relative imaging indices and local hardness (Table S2). X-ray attenuation values generally reflect mineral content and are associated with bone density and mechanical strength [48,49,50]. However, CBCT differs from medical CT in its imaging principles; its gray values are influenced by acquisition conditions and device-specific characteristics, which may limit quantitative accuracy [51]. In this study, no significant correlations were observed between imaging-derived indices and HIT in either cementum or alveolar cortical bone. Several methodological factors might have contributed to the absence of significant associations. First, neither the CBCT nor micro-CT measurements were calibrated using a mineral-density phantom; therefore, the obtained values represented relative imaging indices rather than absolute mineral-density values [52,53]. Second, these indices might have been influenced by scanner-specific acquisition parameters, reconstruction procedures, image noise, and partial-volume effects. Third, the spatial resolution of the imaging modalities differed substantially from the scale of the nanoindentation measurements, particularly for the thin cementum layer. Because the correspondence between individual archived indentation-depth records and specific tissue regions could not be fully reconstructed, and local cementum thickness was not quantitatively measured, the depth-to-thickness ratio could not be verified. Therefore, a potential substrate effect, particularly in thinner cementum regions, cannot be completely excluded. In addition, the limited number of biologically independent donors and the stringent significance threshold applied after Bonferroni correction may have reduced the statistical power to detect weak or moderate correlations between imaging-derived indices and HIT. The small ROI dimensions relative to the spatial sampling characteristics of the imaging modalities may have further limited the sensitivity of the imaging analyses. Therefore, the absence of statistically significant correlations should not be interpreted as evidence of the absence of an association. Accordingly, the present results should not be interpreted as evidence that quantitatively calibrated CT measurements are unrelated to tissue hardness. Rather, they suggest that the uncalibrated relative imaging indices obtained under the present conditions were insufficient to estimate local hardness. These findings expand our understanding of the regional mechanical characteristics of root cementum and adjacent alveolar cortical bone, and highlight the limitations of estimating local tissue hardness from uncalibrated CBCT- and micro-CT-derived imaging indices.

From a biological perspective, root cementum and adjacent alveolar cortical bone are often regarded as structurally continuous mineralized tissues. However, the present findings demonstrate marked heterogeneity in their local mechanical properties at the microscale. Such local differences provide additional insight into the mechanical organization of periodontal tissues; however, their direct clinical significance remains to be determined. Future studies combining imaging, histological evaluation, and mechanical testing will be required to better characterize these tissue properties [28,31,54,55].

This study has several limitations. First, the thickness of human root cementum varies according to anatomical location, generally ranging from approximately 20–50 μm in the cervical region to 150–200 μm in the apical region [56,57].

Second, only seven cadavers were included, and all were elderly (81–94 years old). Although multiple tooth-level measurements were obtained, the number of biologically independent donors remained small. In addition, Specimen 2 had a history of multiple myeloma and contributed a relatively large number of teeth to the dataset. Although no statistically significant differences in HIT were detected among donors, sensitivity analyses excluding Specimen 2 generally yielded results consistent with the primary analyses. The principal findings regarding regional differences in cementum HIT, the higher HIT of central root cementum than adjacent alveolar cortical bone, the associations of region and jaw with cementum HIT, and the lack of significant associations between imaging-derived indices and HIT were retained. However, the comparison between apical root cementum and adjacent alveolar cortical bone changed from non-significant in the primary analysis to statistically significant after exclusion of Specimen 2. Therefore, the present results should be regarded as exploratory and hypothesis-generating rather than confirmatory.

Third, multiple teeth were obtained from the same cadaver. Specimen ID was included as a categorical covariate in the regression models; however, this approach might not have fully accounted for within-cadaver clustering, and pseudo-replication cannot be excluded. Future studies should include a larger number of donors and use multilevel or mixed-effects models.

Fourth, formalin fixation and ethanol storage might have altered the mechanical properties of the tissues. In addition, only indentation hardness was evaluated; elastic modulus, fracture resistance, mineral composition, cementum type, collagen orientation, and microstructural characteristics were not directly examined. Fifth, the CT-derived measurements were not calibrated using a mineral-density phantom; therefore, they represented relative imaging indices rather than absolute mineral-density values. Moreover, the spatial resolution of CBCT and micro-CT differed from the scale of the nanoindentation measurements. Since root cementum is a thin mineralized tissue, partial-volume effects and inclusion of signals from adjacent dentin or surrounding tissues might have affected the imaging measurements. In addition, the imaging ROIs were defined according to their physical dimensions rather than a fixed number of pixels, and their positions relative to the reconstructed image grid were not explicitly standardized. Given the small ROI dimensions relative to the spatial sampling characteristics of the imaging modalities, particularly for CBCT, for which the nominal ROI dimension was smaller than the native in-plane pixel spacing, the resulting indices may have been sensitive to local sampling and ROI placement. Therefore, these values should be interpreted as relative imaging indices rather than quantitative measurements representative of the entire tissue region. In addition, the limited number of biologically independent donors and the stringent significance threshold applied after Bonferroni correction may have reduced the statistical power to detect weak or moderate correlations between imaging-derived indices and HIT. Consequently, the lack of significant correlations observed in this study should not be generalized to quantitatively calibrated, higher-resolution CT protocols.

Finally, although the number of observations included in each analysis is reported in the corresponding tables, the exact number of excluded measurements and the specific reasons for individual exclusions could not be reliably reconstructed retrospectively from the available records. Therefore, a complete quantitative flow of measurements from acquisition to final analysis could not be provided. This limitation should be considered when interpreting the differences in sample size across analyses.

Overall, the present study provides novel insights into the regional mechanical characteristics of human root cementum and adjacent alveolar cortical bone and demonstrates the limitations of estimating local tissue hardness using uncalibrated CT-derived imaging indices, providing a basis for future investigations integrating imaging, histology, and tissue mechanics. Larger studies involving more diverse donors, quantitatively calibrated imaging protocols, higher-resolution acquisition, histological validation, and multilevel statistical analysis are required to confirm these findings.

## 5. Conclusions

In this exploratory study, root cementum exhibited greater hardness in the central than apical regions, whereas adjacent alveolar cortical bone showed no significant regional difference. Under the present acquisition and analysis conditions, uncalibrated CBCT- and micro-CT-derived relative imaging indices were not significantly associated with local hardness. These results suggest that site-specific tissue hardness cannot be inferred from these relative imaging indices alone. The analysis was based on seven elderly cadavers and non-calibrated imaging measurements; hence, the findings should be validated in larger studies using quantitatively calibrated and higher-resolution imaging protocols.

## Figures and Tables

**Table 1:** Baseline characteristics of cadavers and the number of teeth used for each measurement method.

Specimen ID	Sex	Tooth Number	Age (Years)	Cause of Death	Tooth Number (HIT)	Tooth Number (CBCT)	Tooth Number (Micro-CT)
1	Male	16	93	Old age	16	15	16
2	Female	21	81	Multiple myeloma	21	19	20
3	Female	13	91	Multiple organ failure	13	13	13
4	Female	15	94	Old age	15	15	15
5	Female	27	83	Cerebral hemorrhage	26	27	27
6	Female	21	91	Old age	20	21	21
7	Female	21	90	Endometrial cancer	21	20	20

Abbreviations: Indentation hardness (HIT), cone-beam computed tomography (CBCT), computed tomography (CT).

**Table 2:** Comparison of HIT and CT-derived indices between central and apical regions of root cementum and alveolar cortical bone.

			Central		Apical		*p* Value
		*N*	Median	IQR (Q1–Q3)	Median	IQR (Q1–Q3)	
Cementum	HIT (MPa)	115	205.8	133.6–272.1	157.7	102.9–226.5	<0.001
	Maximum CBCT GV	116	368.5	254.3–540.3	320.0	235.3–524.8	<0.001
	Mean CBCT GV	116	355.1	237.8–528.2	297.8	211.8–487.3	<0.001
	Maximum micro-CT GV	127	448.0	307.0–596.0	355.0	192.0–491.0	<0.001
	Mean micro-CT GV	127	344.8	231.0–513.0	264.3	134.3–407.5	<0.001
Alveolar cortical bone	HIT (MPa)	100	107.8	74.8–161.2	113.0	92.5–157.1	0.283
	Maximum CBCT GV	108	273.5	187.8–467.0	235.0	163.5–414.5	0.003
	Mean CBCT GV	107	241.3	165.3–443.8	214.5	149.0–359.5	0.004
	Maximum micro-CT GV	108	259.5	60.8–397.3	137.0	−45.3–345.0	0.004
	Mean micro-CT GV	106	134.5	−56.3–312.6	16.5	−117.7–241.4	0.010

Abbreviations: Gray value (GV), inter-quartile range (IQR), quartile 1–3 (Q1–Q3); N represents the number of matched pairs included in the Wilcoxon signed-rank test.

**Table 3:** Comparison of HIT and CT-derived indices between root cementum and alveolar cortical bone in the central and apical regions.

			Cementum		Alveolar Cortical Bone		*p* Value
		*N*	Median	IQR (Q1–Q3)	Median	IQR (Q1–Q3)	
Central	HIT (MPa)	108	206.6	134.3–282.7	107.8	80.8–156.0	<0.001
	Maximum CBCT GV	110	372.0	254.8–552.0	273.5	185.8–474.0	<0.001
	Mean CBCT GV	109	356.0	238.5–530.4	241.3	164.6–449.9	<0.001
	Maximum micro-CT GV	116	446.0	304.8–585.3	244.0	56.5–394.0	<0.001
	Mean micro-CT GV	116	336.4	214.9–508.3	121.5	−70.6–300.7	<0.001
Apical	HIT (MPa)	106	162.2	117.0–226.7	113.4	92.2–155.5	0.299
	Maximum CBCT GV	116	322.0	235.3–523.0	241.0	175.5–395.3	<0.001
	Mean CBCT GV	116	299.6	217.6–494.8	216.8	152.3–358.9	<0.001
	Maximum micro-CT GV	118	348.0	193.5–485.5	142.5	−42.3–338.3	<0.001
	Mean micro-CT GV	117	257.1	135.5–415.5	15.7	−119.8–233.9	<0.001

N represents the number of matched pairs included in the Wilcoxon signed-rank test.

**Table 4:** Multiple linear regression analysis of factors associated with HIT in root cementum and alveolar cortical bone.

Independent Variable		Cementum (*N* = 120)			Alveolar Cortical Bone (*N* = 110)		
		B	95% CI for B	*p* Value	B	95% CI for B	*p* Value
Region	Central	42.6	13.3–71.9	0.005	23.3	−44.3–90.9	0.498
	Apical		Reference			Reference	
Specimen ID	1	4.6	−51.5–60.7	0.872	−86.1	−213.6–41.3	0.184
	2	43.3	−5.1–91.6	0.079	−31.2	−144.2–81.7	0.586
	3	56.8	−0.2–113.9	0.051	−80.8	−210.5–48.8	0.220
	4	48.5	−5.6–102.7	0.079	−16.0	−143.8–111.7	0.805
	6	−4.2	−55.3–46.9	0.827	−57.9	−183.9–68.2	0.366
	7	−19.0	−66.8–28.7	0.433	−65.4	−176.0–45.1	0.244
Jaw region	Maxilla	−31.6	−61.3 to −1.9	0.037			
	Mandible		Reference				
Presence of metal prostheses	No				26.3	−57.5–110.1	0.537
	Yes					Reference	
History of root canal treatment	No	−5.3	−42.3–31.7	0.777	−18.4	−106.9–70.1	0.683
	Yes		Reference			Reference	
Adjusted *R*^2^			0.066			−0.023	
Model *p* value			0.003			0.902	

Abbreviation: Confidence interval (CI); N represents the number of measurement-site observations included in the regression model. Only observations meeting the matching criteria for the central and apical regions were included in the analysis.

## Data Availability

The data presented in this study are available from the corresponding author upon reasonable request.

## Supplementary Materials

The following supporting information can be downloaded at: https://www.mdpi.com/article/10.3390/dj14100670/s1, Table S1: Univariate analysis of factors associated with hardness (HIT) in root cementum and alveolar cortical bone.; Table S2: Correlation between indentation hardness (HIT) and CT-derived indices in root cementum and alveolar cortical bone; Table S3: Sensitivity analysis comparing the primary analysis with the analysis excluding Specimen 2.

## Abbreviations

The following abbreviations are used in this manuscript:

CBCT	Cone-beam computed tomography
Micro-CT	Micro-computed tomography
HIT	Indentation hardness
CEJ	Cementoenamel junction
ROI	Region of interest
