# Supplementary material for: Site-Specific Nanoindentation Hardness of Human Root Cementum and Corresponding Alveolar Cortical Bone: Associations with Uncalibrated CBCT and Micro-CT Gray-Value Indices
Source: Dent J (Basel). 2026 Oct 14;14(10):670. doi: 10.3390/dj14100670 (PMC13703185; doi:10.3390/dj14100670)
Supplement: Supplementary file 1 [file dentistry-14-00670-s001.zip › dentistry-4568032-supplementary.pdf]

Table S1. Univariate analysis of factors associated with hardness (HIT) in root cementum and alveolar cortical bone

		Cementum								Alveolar cortical bone							
		Central (*N* = 120)				Apical (*N* = 124)				Central (*N* = 112)				Apical (*N* = 110)			
		N	Median	IQR (Q1–Q3)	*P* value	*N*	Median	IQR (Q1–Q3)	*P* value	*N*	Median	IQR (Q1–Q3)	*P* value	*N*	Median	IQR (Q1–Q3)	*P* value
Specimen ID	1	12	244.3	125.2–338.7	0.349	13	153.8	84.3–226.7	0.168	12	100.7	71.6–119.5	0.247	12	109.4	82.4–135.4	0.174
	2	20	226.1	160.8–274.7		20	182.0	138.0–241.7		20	117.4	74.0–192.8		18	126.0	101.7–226.8	
	3	12	247.7	182.8–353.6		12	196.8	95.7–330.7		12	108.7	83.8–158.1		11	91.4	56.8–141.8	
	4	13	207.3	146.8–268.3		15	178.7	120.1–249.8		13	136.4	97.6–283.8		12	116.1	105.9–170.5	
	5	26	173.6	77.5–286.6		25	163.1	121.7–201.8		23	111.1	65.8–179.5		22	108.6	56.2–129.5	
	6	16	168.8	142.7–204.3		18	148.5	101.6–198.2		12	124.7	96.5–154.2		15	125.7	92.3–188.5	
	7	21	167.5	114.2–254.4		21	117.6	86.2–191.6		20	93.1	69.1–109.4		20	120.1	100.6–209.9	
Jaw region	Maxilla	61	170.4	114.3–250.7	0.027	64	149.2	98.0–219.7	0.204	54	99.4	75.1–145.9	0.403	55	119.5	94.2–157.4	0.270

	Mandible	59	218.1	159.0–323.9		60	165.7	120.2–227.9		58	110.3	82.7–177.4		55	107.9	88.2–149.1	
Tooth type	Anterior	65	183.8	132.9–260.7	0.292	66	155.6	103.9–210.5	0.618	60	110.3	82.6–150.7	0.618	58	112.2	94.3–134.3	0.272
	Posterior	55	211.0	144.4–297.4		58	165.7	102.7–229.7		52	102.8	69.4–169.6		52	115.0	90.9–229.9	
Presence of metal prostheses	No	87	191.8	126.6–272.1	0.438	88	157.9	106.9–221.4	0.936	79	102.4	76.2–150.3	0.400	76	110.2	90.8–138.3	0.039
	Yes	33	208.6	154.3–281.8		36	156.9	100.5–226.2		33	117.4	89.3–177.4		34	125.7	95.3–250.7	
Presence of opposing tooth	No	17	243.7	138.3–351.1	0.251	16	184.9	80.1–287.1	0.693	16	101.3	86.1–153.8	0.784	14	100.0	76.1–333.5	0.635
	Yes	103	201.8	133.6–266.2		108	156.5	106.9–217.5		96	108.7	76.9–168.1		96	115.4	92.9–149.0	
History of root canal treatment	No	96	207.2	132.2–285.3	0.935	96	165.7	110.1–228.1	0.162	87	102.4	79.1–150.3	0.472	84	108.6	90.6–148.6	0.087
	Yes	24	204.5	140.4–252.7		28	141.2	95.2–194.9		25	136.4	75.5–176.5		26	128.3	105.1–197.9	

Abbreviations: IQR, interquartile range; Q1, first quartile; Q3, third quartile.; *P* values were calculated using the Mann–Whitney U test for comparisons between two groups and the Kruskal–Wallis test for comparisons among specimen IDs. *P* values < 0.05 are shown in bold.; N represents the number of tooth-level observations included in each analysis.

Table S2. Correlation between indentation hardness (HIT) and CT-derived indices in root cementum and alveolar cortical bone

HIT			Maximum CBCT GV	Mean CBCT GV	Maximum micro-CT GV	Mean micro-CT GV
Cementum	Central	r_s	−0.106	−0.093	−0.002	0.012
		P	0.272	0.332	0.981	0.895
		N	110	110	117	117
	Apical	r_s	−0.176	−0.172	−0.127	−0.128
		P	0.064	0.071	0.167	0.163
		N	111	111	120	120
Alveolar cortical bone	Central	r_s	−0.175	−0.194	−0.213	−0.235
		P	0.088	0.060	0.033	0.018
		N	96	95	100	100
	Apical	r_s	0.069	0.085	0.050	0.045
		P	0.500	0.407	0.620	0.654
		N	97	97	102	100

Abbreviations: HIT, indentation hardness; CBCT, cone-beam computed tomography; micro-CT, micro-computed tomography; GV, gray value; *r*s, Spearman's rank correlation coefficient.; N represents the number of tooth-level observations with available paired HIT and imaging-derived index data included in each correlation analysis.; Correlations were assessed using Spearman's rank correlation analysis. Statistical significance was determined using the Bonferroni-corrected significance level of $P < 0.003125$.

Table S3. Sensitivity analysis comparing the primary analysis with the analysis excluding Specimen 2

Outcome	Primary analysis, 7 donors	Excluding Specimen 2, 6 donors	Interpretation
Cementum HIT: central vs apical	205.8 (133.6–272.1) vs 157.7(102.9–226.5), *P* < 0.001	192.7 (128.0–271.6) vs 155.0 (102.9–210.7), *P* < 0.001	Maintained
Alveolar bone HIT: central vs apical	107.8 (74.8–161.2) vs 113.0(92.5–157.1), *P* = 0.283	105.8 (79.9–156.8) vs 112.2 (90.8–142.7), *P* = 0.447	Maintained
Cementum vs alveolar bone: central	206.6 (134.3–282.7) vs 107.8 (80.8–156.0), *P* < 0.001	192.7 (128.0–271.6) vs 105.7 (79.9–156.8), *P* < 0.001	Maintained
Cementum vs alveolar bone: apical	162.2 (117.0–226.7) vs 113.4(92.2–155.5), *P* = 0.299	155.0 (102.9–210.8) vs 111.7 (90.7–143.0), *P* < 0.001	Changed
Cementum regression: central region	B = 42.6 (13.3–71.9), *P* = 0.005	B = 47.4 (16.1–78.6), *P* = 0.003	Maintained
Cementum regression: jaw	B = −31.6 (−61.3 to −1.9), *P* = 0.037	B = −36.2 (−68.0 to −4.5), *P* = 0.026	Maintained
HIT-CBCT/micro-CT correlations	No significant associations	No significant associations	No material change

Abbreviations: HIT, indentation hardness; CBCT, cone-beam computed tomography; CI, confidence interval.; Data are presented as medians for group comparisons and as unstandardized regression coefficients (B) with 95% confidence intervals (CI) for multivariable regression analyses. P values for paired comparisons were obtained using the Wilcoxon signed-rank test. Correlations between HIT and CBCT/micro-CT indices were assessed using Spearman's rank correlation coefficient (r_s), with Bonferroni correction for multiple comparisons (adjusted significance level, $P < 0.003125$). Specimen 2 was excluded from the sensitivity analyses because of a history of multiple myeloma and its relatively large contribution of teeth to the dataset.
